# The long-term impact of the COVID-19 pandemic on physical fitness in young adults: a historical control study

**DOI:** 10.1038/s41598-023-42710-0

**Published:** 2023-09-18

**Authors:** Jeffrey W. Ripley-Gonzalez, Nanjiang Zhou, Tanghao Zeng, Baiyang You, Wenliang Zhang, Jie Liu, Yuchen Dong, Ying Guo, Yaoshan Dun, Suixin Liu

**Affiliations:** 1 Division of Cardiac Rehabilitation, Department of Physical Medicine & Rehabilitation, Xiangya Hospital of Central South University, Changsha, Hunan China; 2 National Clinical Research Center for Geriatric Disorders, Xiangya Hospital of Central South University, Changsha, Hunan China; 3 Hunan Traditional Chinese Medical College, Zhuzhou, Hunan China; 4 Medical College of Jinhua Polytechnic, Jinhua, Zhejiang China; 5 Division of Preventive Cardiology, Department of Cardiovascular Medicine, Mayo Clinic, Rochester, MN USA

**Keywords:** Public health, Health policy

## Abstract

The strength of evidence regarding long-term changes to fitness resulting from the coronavirus disease 2019 (COVID-19) lockdowns is deficient. This two-site retrospective study aimed to investigate the long-term changes in physical fitness among young adults a year after the onset of the pandemic using a robust historical control. University freshmen who underwent physical fitness tests in 2019 and completed a follow-up in 2020 (study group) were included. The primary focus was to compare the current cohort with a historical control group who completed the same tests a year prior (2018). A total of 5376 individuals were recruited, of which 2239 were in the study group. Compared with the control, the study group exhibited a decrease in anaerobic fitness, with an overall difference of −0.84 (95% confidence interval [CI], [−1.33 to −0.36]); declines in aerobic fitness, with a difference of −2.25 [−3.92 to −0.57] for males and −4.28 [−4.97 to −3.59] for females; a reduced explosive fitness (−2.68 [−3.24 to −2.12]); and a decreased upper-body strength in females (−1.52 [−2.16 to −0.87]). The fitness of young adults has been considerably compromised by COVID-19 lockdowns, highlighting the importance of promoting physical activity to prevent long-term health implications.

## Introduction

Physical fitness plays a crucial role in maintaining overall health and well-being and is associated with a range of health benefits, including a reduced risk of cardiovascular disease and metabolic disorders and lower overall mortality rates^1,2^. In view of these, multinational guidelines recommend regular physical activity and exercise across all age groups, while also highlighting the need to limit sedentary behaviours^3^.

However, the coronavirus disease 2019 (COVID-19) pandemic and the accompanying measures implemented to mitigate its spread have posed significant challenges in maintaining optimal physical fitness levels, leading to increased sedentary behaviour and decreased exercise participation among many individuals^4,5^. Multiple published research have reported on the wide-ranging effects of lockdowns. For example, our previous research has documented acute self-reported weight gain, reduced overall physical activity and exercise, and increased sedentary behaviour, which aligns with findings reported elsewhere during the pandemic^6,7^. Additionally, our research has revealed an increase in psychological issues associated with COVID-19 mitigation measures affecting young individuals^8^. It is worth noting that these effects might have been influenced by baseline fitness levels^9^. However, there is a limited body of research exploring the long-term effects of the pandemic on physical fitness. While some studies have investigated the effects of COVID-19 on physical fitness in children^10^, research focusing on standardised fitness testing in adult populations is scarcer due to insufficient data. Moreover, most studies that have examined this topic have relied on subjective fitness questionnaires to assess acute changes^11,12^. Although qualitative research has provided valuable insights into how individuals’ behaviours might be affected by the pandemic and lockdowns, it falls short of providing an accurate representation of fitness changes compared with objective measurements. Recent research by Yu et al. suggested that changes in physical activity over time could be associated with decreased fitness after the pandemic, however, this study only offers a snapshot in time as it lacks baseline data from before the pandemic^13^. Lastly, a prevalent issue in the existing evidence is the lack of control groups in studies involving young adult populations, significantly limiting the strength of the evidence^14,15^.

The current study aimed to investigate the long-term changes in objectively measured fitness parameters among young adults, a year after the onset of the COVID-19 pandemic to address this gap in the literature. A robust historical control group was used to evaluate these changes, allowing us to make inferences regarding the effect of the COVID-19 lockdowns by comparing the study group to a similar group of individuals who were unaffected by COVID-19 lockdowns. This novel approach will allow for a comparison between groups which can contribute to a more complete understanding of the long-term impact of the COVID-19 pandemic and aid in developing effective measures against future epidemics.

## Materials and methods

### Study design and participants

From December 2019 to January 2021, data collection was conducted at two centres (in Central and Central-East China, respectively) to assess the longitudinal changes in the physical fitness of university-aged students. Participants were recruited from the Chinese Medical College, Hunan, China, and the Medical College of Jinhua Polytechnic, Zhejiang, China. These institutions conducted the inaugural Chinese National Student Physical Fitness Standard (CNSPFS) battery between 1 December 2019 and 20 January 2020 before the implementation of national lockdowns. After a year, these participants were followed up, and the CNSPFS battery was administered again between 1 December 2020 and 20 January 2021. At the time of enrolment all participants involved in this study had begun their first year of higher education, with a mean age of 18 (standard deviation (SD): 1). Participants were excluded from physical fitness tests if they had a pre-existing medical condition which would impede their ability to perform exercise safely.

Baseline data was retrieved from the CNSPFS system, and data from both time points were linked using each participant's university student identity number. This research was approved by the Institutional Review Board of Xiangya Hospital of Central South University (approval no. 202005126). Written informed consent was obtained from all participants, and the study was performed in accordance with the principles of the Declaration of Helsinki. This manuscript follows the reporting guidelines set by STROBE.

### Historical control

A group was required for comparison to fully understand the effect of the pandemic on objective measurements of fitness and weight changes. The historical control group was established by obtaining the physical fitness records of students who had enrolled in the same two universities in 2018 and had completed the CNSPFS battery a year prior to the study. The first visit of the historical control group was between 1 December 2018 and 20 January 2019, while their second visit was between 1 December 2019 and 20 January 2020, before the implementation of nationwide lockdowns. This control group was selected to provide a baseline for comparing the findings with current study participants.

### Outcomes

The primary outcomes were measured in an open-air track field, which included changes in several performance and fitness scores. These scores were derived from various tests, including a 50-m sprint^16^, an 800-m run for females and a 1000-m run for males^17^, a standing long jump^18^, timed 1-min sit-ups for females and pull-ups for males^19^, a sit and reach test, and vital lung capacity measurement^20^. These tests assessed various aspects of fitness, such as anaerobic capacity, aerobic endurance, explosive power, muscular strength, flexibility, and pulmonary function.

The CNSPFS was conducted according to the standard operating procedures, under the supervision of trained physical education teachers. The assessment commenced with the collection of anthropometric data, including height measured using a portable stadiometer and weight measured using an electronic weight scale. Body mass index (BMI) was then calculated for each participant by dividing their weight in kilograms by the square of their height in meters. All physical fitness tests were conducted in the track-fields within the grounds of each respective university. Throughout the COVID-19 period, all students had to take bi-weekly nucleic acid testing as part of the “zero-COVID” governmental policy. During the testing period, there were no COVID-19 infections amongst the participant populations or testing staff of either university. As a result all tests were conducted as scheduled.

Secondary outcomes included the frequency of aerobic and strength training (prior to the lockdown, during the lockdown, and at the 2-year follow-up (1 year after the second CNSPFS visit) in the study group. Daily sedentary time and computer usage were recorded at three time points: during the first CNSPFS assessment, during the lockdown and a year after the second CNSPFS assessment).

### Standardisation of the CNSPFS battery

Physical fitness measurements were obtained by administering the CNSPFS battery and the scores were calculated using a nationally standardised scoring system that adjusted each fitness indicator score for age- and sex-specific percentages. The scores were categorised into four groups: low fitness (< 60), moderate fitness (60–79), high fitness (80–89), and excellent fitness (≥ 90). An intraclass correlation coefficient of > 0.90 was achieved to ensure consistency between assessments. The details regarding performing CNSPFS have been described previously ^21,22^.

### Sample size

Based on previous research^23^ that reported a mean difference between groups in the change of total fitness score of 2.84, with a standard deviation of 9.3, we conducted a sample size calculation via a two-sided two-sample t-test with an alpha level at 0.05 and a power of 0.90. PASS version 15.0.5 software (Utah, USA) was used to calculate. The estimated required sample size was 456 participants, with 228 per group. Anticipating a 20% failure to attain complete physical testing data, a minimum of 570 participants were required, which was considerably less than was finally attainable for this research study.

### Statistical analysis

The normality of continuous variables was assessed using the Shapiro–Wilk test. Normally distributed continuous variables are presented as means ± SD, while non-normally distributed variables are presented as the median (interquartile range). Categorical variables are presented as numbers (percentages). Baseline data were compared using independent samples *t*-test and the chi-square test. For the primary outcomes and secondary outcomes analyses, linear mixed models were pre-specified. The models adjusted for schools, age, sex, location, regional disposable income, and the value of the outcome and at baseline differences. Subgroup analyses were conducted for male and female, urban and rural subgroups to examine the consistency of the primary outcome across different areas. Statistical significance was set at *p* < 0.05. All statistical analyses were performed using SPSS 26.0 (IBM Inc., Chicago, USA).

### Ethical approval

The study was conducted in accordance with the Declaration of Helsinki, and approved by the Institutional Review Board of Xiangya Hospital of Central South University (approval no. 202005126).

### Informed consent

Written informed consent was documented during the baseline, and digital informed consent was given upon initiating the survey.

## Results

### Demographics

**Figure 1:**
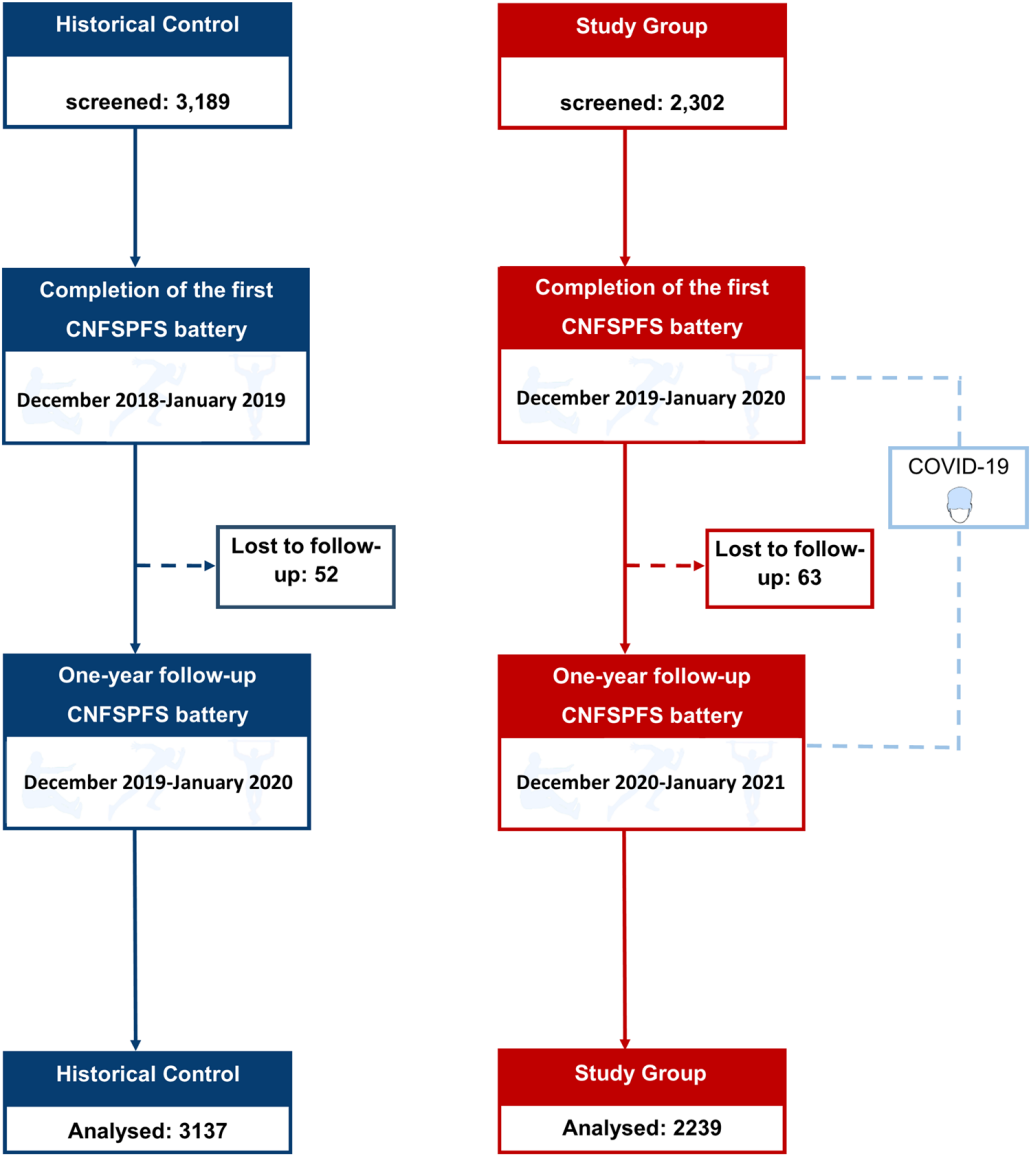
Flow chart showing the study processes of both groups. Chinese National Student Physical Fitness Standard (CNSPFS).

A total of 5376 individuals were included in the analysis, with 2239 participants in the study group and 3137 participants in the control group, recruited from two universities. The study flow chart is presented in Fig. 1. The baseline demographics of both groups are presented in Table 1. Female participants constituted a higher proportion of the population. In the study group, 61.5% of the participants resided in urban areas, while 38.5% resided in rural communities. In the historical control group, these percentages were 64.5% and 35.5%, respectively. Age in both groups was a median of 18 years (interquartile range (IQR) of 18–19), with a mean age of 18 years (SD:1) in both groups (*P* = 0.45*)*. No differences were observed in baseline height, weight, or socioeconomic conditions between the two groups. Furthermore, no significant difference was observed at baseline for BMI, with the Study group mean BMI of 20.6 kg/m^2^ (SD: 3) and Historical Control of 20.7 kg/m^2^ (SD: 3), median (IQR) of 20.0 kg/m^2^ (18.6–22) in the study group and 20.1 kg/m^2^ (18.7–22) in the historical control. However, significant differences were observed between the groups in terms of baseline sit-and-reach tests and 1-min sit-ups.

**Table 1:** Baseline characteristics.

	Study group	Historical control	*P* value
	N = 2239	N = 3137	
University			
Zhuzhou	1785 (79.7%)	2436 (77.7%)	0.07
Jinhua	454	701	
Sex			0.002
Female	1910 (85.3%)	2577 (82.1%)	
Location			
Urban	1378 (61.5%)	2024 (64.5%)	0.03
Rural	861	1113	
Regional disposable income (per capita)	28,646 (13,714)	29,162(14,009)	0.18
Age	18 (1)	18 (1)	0.26
Height (cm)	161 (7)	162 (7)	0.24
Weight (kg)	53.8 (9.1)	54.2 (9.5)	0.18
BMI (kg/m^2^)	20.6 (3)	20.7 (3)	0.45
Vital capacity (ml)	2911 (623)	3059 (661)	< 0.001
50 m run (s)	8.96 (0.89)	8.98 (0.93)	0.26
Anaerobic fitness	71 (10)	70 (10)	0.001
Standing long jump (cm)	176.5 (25.1)	176 (25.8)	0.46
Explosive fitness	69 (14)	66 (16)	< 0.001
Sit and reach test (cm)	16.44 (5.51)	16.07 (6.09)	0.02
Flexibility	76 (12)	75 (15)	0.001
Male	N = 329	N = 560	
1000 m run (s)	247.79 (24.15)	251.06 (32.45)	0.09
Aerobic fitness	63 (13)	61 (16)	0.06
One-minute pull-ups	9.00 (5.38)	8.26 (5.68)	0.33
Muscular strength	46 (33)	43 (34)	0.15
Female	N = 1910	N = 2577	
800 m run (s)	239.69 (19.18)	237.36 (24.39)	< 0.001
Aerobic fitness	67 (12)	67 (15)	0.20
One-minute sit-ups	33.62 (7.52)	31.72 (8.82)	< 0.001
Muscular strength	66 (13)	61 (18)	< 0.001
Total score	69 (8)	67 (10)	< 0.001

BMI, body mass index; SD, standard deviation. Data are expressed as mean (SD) or numbers (percentages) accordingly. Independent samples t-test for continuous distributed data and the chi-square test for categorical variables between groups. The score is based on grading standards for Chinese university students. The total score is the mean of all other fitness scores, for males and females. *P* < 0.05 is considered statistically significant.

### Change in fitness

**Figure 2:**
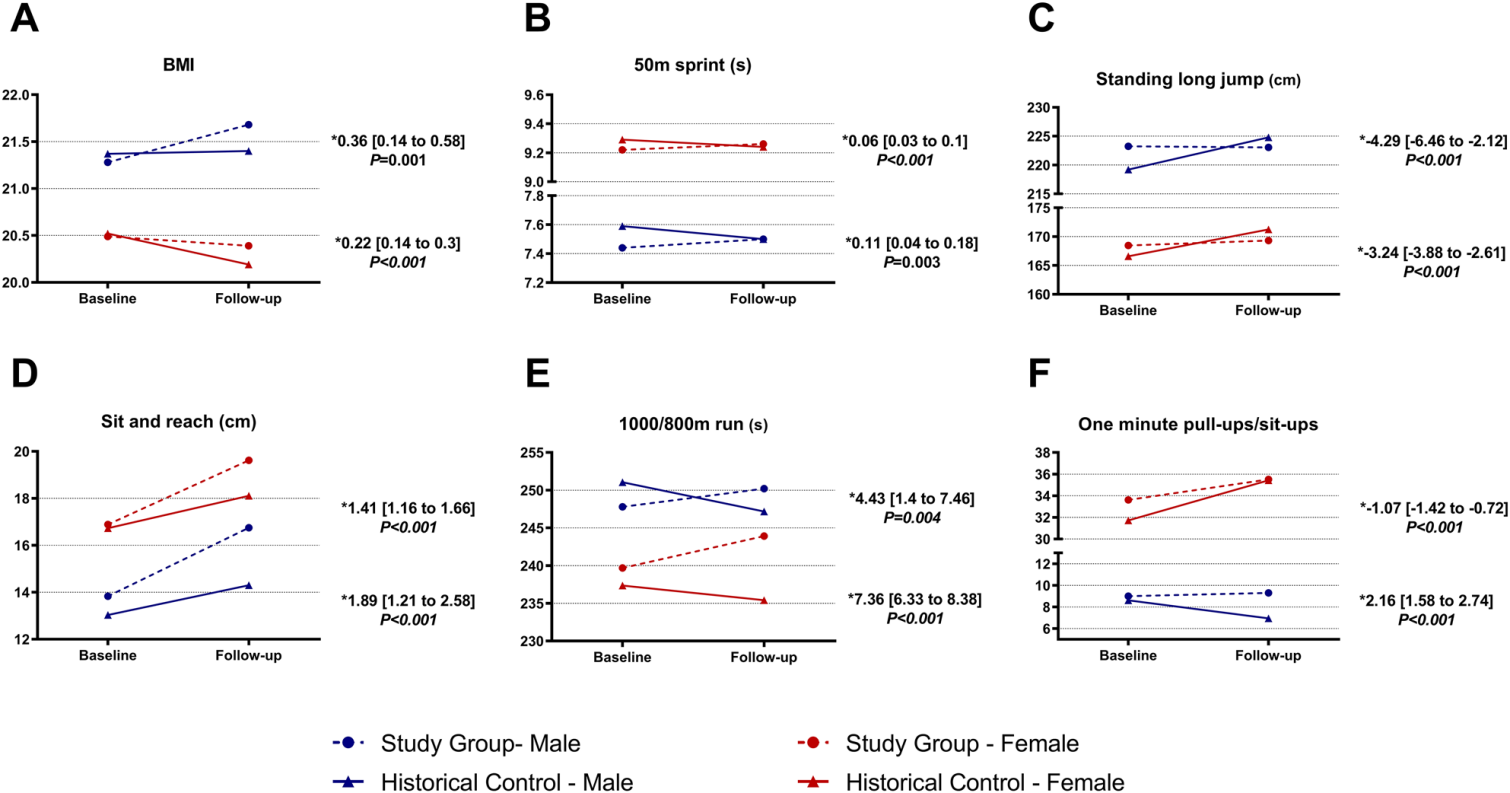
Changes in physical fitness performance within each group. BMI, body mass index. (*) used to show the confidence interval. G, Female participants completed an 800 m run, and Male participants completed a 1000 m run. H, Female participants’ measure of strength was the maximum number of sit-ups completed in a single minute, for male participants this was the maximum number of pull-ups completed in one minute.

**Figure 3:**
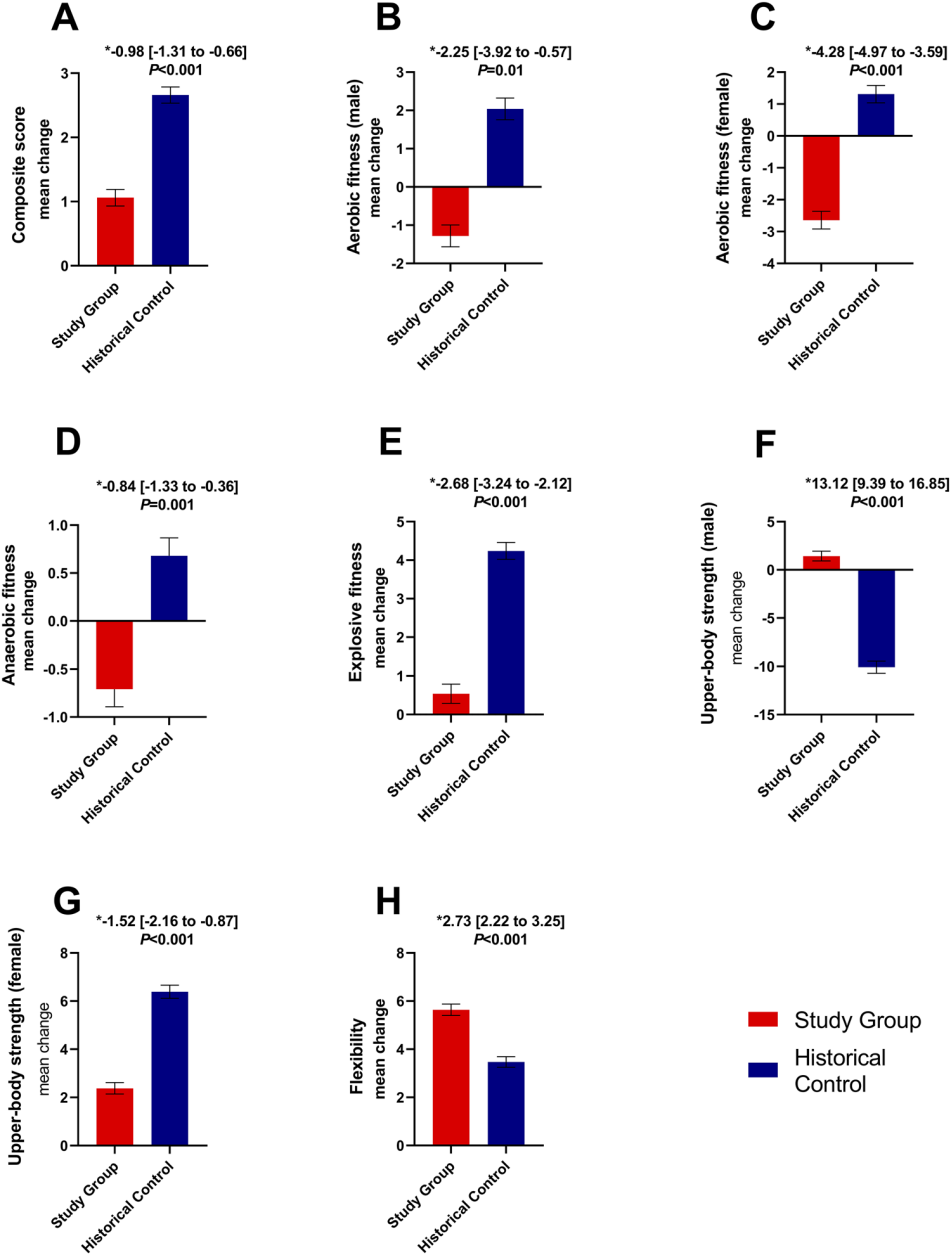
Changes in physical fitness between study and historical groups. (*) used to show the confidence interval. Fitness was weighed according to the standardised system that weighted each fitness indicator score by age- and sex-specific percentage. Scores were grouped into: low fitness (below 60), moderate fitness (60 to 79), high fitness (80 to 89), and excellent fitness (90 and above). The consistency between assessments was ensured through an intraclass correlation coefficient (ICC) that was greater than 0.90. The graph shows mean changes in scores: (**A**) Total composite score; (**B**) Aerobic score (males); (**C**) Aerobic score (females); (**D**) Anaerobic score; (**E**) Explosive fitness (score); (**F**) Upper-body strength (male); (**G**) Upper-body strength (female); (**H**) Flexibility.

The changes in fitness measures between the study group and the historical control group are presented in Table 2, Figs. 2 and 3. Figure S1 illustrates the changes in fitness as proportions of the population. The statistical results remained consistent across subgroup analysis (Supplementary Table S1).

**Table 2:** Changes before and one year after the pandemic and difference between the two groups.

Total	Study group			Historical control				
	N = 2239			N = 3137				
	Baseline mean (SD)	After	Mean change (SD)	Baseline mean (SD)	After	Mean change (SD)	Mean Difference [95%CI]	*P* value
Height (cm)	161 (7)	162 (7)	0.09 (1.46)	162 (7)	162 (7)	0.66 (1.46)	−0.55 [−0.63 to −0.48]	< 0.001
Weight (kg)	53.8 (9.1)	53.8 (9.6)	0 (3.9)	54.1 (9.5)	53.9 (9.7)	−0.24 (3.8)	0.29 [0.09 to 0.50]	0.004
BMI (kg/m^2^)	20.6 (3)	20.6 (3.1)	−0.03 (1.5)	20.7 (3)	20.4 (3)	−0.3 (1.47)	0.24 [0.17 to 0.32]	< 0.001
Vital capacity (ml)	2911 (623)	2998 (661)	87 (465)	3059 (661)	3085 (669)	26 (392)	19.72 [−0.48 to 39.91]	0.06
50 m (s)	8.96 (0.89)	9 (0.91)	0.04 (0.53)	8.98 (0.93)	8.93 (0.97)	−0.05 (0.6)	0.07 [0.04 to 0.1]	< 0.001
Anaerobic fitness	71 (10)	70 (11)	−0.7 (9)	70 (10)	71 (11)	0.7 (11)	−0.84 [−1.33 to −0.36]	0.001
Standing long jump (cm)	176.5 (25.1)	177.2 (25.2)	0.7 (13.2)	176 (25.8)	180.8 (26.1)	4.8 (12.3)	−3.43 [−4.07 to −2.79]	< 0.001
Explosive fitness	69 (14)	69 (14)	0.5 (12)	66 (16)	70 (14)	4 (12)	−2.68 [−3.24 to −2.12]	< 0.001
Sit and reach (cm)	16.4 (5.51)	19.2 (5.69)	2.8 (5.05)	16.07 (6.09)	17.43 (5.68)	1.36 (4.75)	1.5 [1.26 to 1.73]	< 0.001
Flexibility	76 (12)	82 (11)	6 (11)	75 (15)	78 (12)	4 (13)	2.73 [2.22 to 3.25]	< 0.001
Total score	69 (8)	70 (8)	1 (6)	67 (10)	70 (9)	3 (7)	−0.98 [−1.31 to −0.66]	< 0.001

Male	Study group			Historical control				
	N = 329			N = 560				
	Baseline mean (SD)	After	Mean change (SD)	Baseline mean (SD)	After	Mean change (SD)	Mean difference [95%CI]	*P* value
Height (cm)	172 (6)	172 (6)	0.2 (1.6)	172 (6)	173 (6)	0.8 (1.6)	−0.56 [−0.77 to −0.35]	< 0.001
Weight (kg)	62.9 (10.6)	64.2 (11.9)	1.3 (5.3)	63.22 (11.2)	63.8(11.3)	0.6 (4.3)	0.71 [0.07 to 1.36]	0.029
BMI (kg/m^2^)	21.28 (3.37)	21.68 (3.79)	0.39 (1.85)	21.37 (3.55)	21.4 (3.61)	0.03 (1.48)	0.36 [0.14 to 0.58]	0.001
Vital capacity (ml)	3940 (579)	4000 (670)	61 (606)	4111 (590)	4147 (628)	36 (456)	−11.78 [−77.95 to 54.4]	0.73
50 m (s)	7.44 (0.58)	7.5 (0.64)	0.06 (0.51)	7.59 (0.53)	7.5 (0.68)	−0.09 (0.55)	0.11 [0.04 to 0.18]	0.003
Anaerobic fitness	79 (11)	78 (12)	−1 (10)	76 (9)	78 (11)	2 (9)	−2.13 [−3.37 to −0.89]	0.001
Standing long jump (cm)	223.3 (20.5)	223.1 (22)	−0.2 (19.6)	219.2 (20.8)	224.8 (20.9)	5.6 (15.5)	−4.29 [−6.46 to −2.12]	< 0.001
Explosive fitness	64 (18)	63 (20)	−0.8 (19)	59 (20)	64 (19)	5 (16)	−3.37 [−5.45 to −1.28]	0.002
Sit and reach (cm)	13.8 (5.6)	16.8 (6.1)	2.9 (5.9)	13 (7)	14.3 (6.4)	1.3 (5.7)	1.89 [1.21 to 2.58]	< 0.001
Flexibility	73 (13)	79 (11)	6 (13)	71 (18)	74 (14)	4 (15)	3.72 [2.22 to 5.21]	< 0.001
1000 m run (s)	247.8 (24.2)	250.2 (25)	2.4 (22.9)	251 (32.5)	247.2 (25.9)	−3.9 (30.4)	4.43 [1.4 to 7.46]	0.004
Aerobic fitness	63 (13)	62 (14)	−1.3 (14)	61 (16)	63 (14)	2 (16)	−2.25 [−3.92 to −0.57]	0.01
One−minute pull−ups (repetitions)	9 (5.4)	9.3 (5.2)	0.3 (3.8)	8.6 (5.7)	7 (5.1)	−1.7 (5.6)	2.16 [1.58 to 2.74]	< 0.001
Muscular strength	46 (33)	48 (33)	1 (24)	43 (34)	33 (32)	−10 (36)	13.12 [9.39 to 16.85]	< 0.001
Total score	65 (12)	66 (12)	1 (9)	62 (12)	62 (12)	0.4 (10)	1.46 [0.29 to 2.63]	0.02

Female	Study group			Historical control				
	N = 1910			N = 2577				
	Baseline mean (SD)	After	Mean change (SD)	Baseline mean (SD)	After	Mean change (SD)	Mean difference [95%CI]	*P* value
Height (cm)	160 (5)	160 (5)	0.07 (1.44)	159 (5)	160 (5)	0.64 (1.44)	−0.55 [−0.64 to −0.47]	< 0.001
Weight (kg)	52.2 (7.82)	52 (7.9)	−0.23 (3.55)	52.2 (7.9)	51.8 (7.8)	−0.43 (3.62)	0.21 [0.01 to 0.42]	0.04
BMI (kg/m^2^)	20.49 (2.86)	20.39 (2.9)	−0.11 (1.44)	20.52 (2.82)	20.19 (2.78)	−0.33 (1.46)	0.22 [0.14 to 0.3]	< 0.001
Vital capacity (ml)	2734 (429)	2826 (482)	92 (436)	2830 (405)	2854 (400)	24 (377)	24.58 [4.22 to 44.94]	0.02
50 m run (s)	9.22 (0.64)	9.26 (0.66)	0.04 (0.53)	9.29 (0.68)	9.24 (0.71)	−0.05 (0.61)	0.06 [0.03 to 0.1]	< 0.001
Anaerobic fitness	70 (9)	69 (10)	−0.6 (8)	69 (10)	69 (11)	0.4 (11)	−0.64 [−1.17 to −0.12]	0.02
Standing long jump (cm)	168.5 (14.9)	169.3 (15.4)	0.9 (11.7)	166.6 (14.9)	171.2 (15)	4.7 (11.5)	−3.24 [−3.88 to −2.61]	< 0.001
Explosive fitness	70 (13)	70 (12)	0.8 (10)	68 (15)	72 (12)	4.2 (11.3)	−2.54 [−3.08 to −2]	< 0.001
Sit and reach (cm)	16.9 (5.4)	19.6 (5.5)	2.7 (4.9)	16.7 (5.7)	18.1 (5.3)	1.4 (4.5)	1.41 [1.16 to 1.66]	< 0.001
Flexibility	77 (12)	82 (11)	6 (11)	76 (14)	79 (11)	3 (12)	2.52 [1.98 to 3.07]	< 0.001
800 m run (s)	239.7 (19.2)	243.9 (20.9)	4.2 (17.5)	237.4 (24.4)	235.4 (19.7)	−2 (23.3)	7.36 [6.33 to 8.38]	< 0.001
Aerobic fitness	67 (12)	64 (14)	−3 (13)	67 (15)	68 (12)	1 (15)	−4.28 [−4.97 to −3.59]	< 0.001
One-minute sit-ups (repetitions)	33.6 (7.5)	35.5 (8.1)	1.9 (6.2)	31.7 (8.8)	35.4 (8.2)	3.7 (6.9)	−1.07 [−1.42 to −0.72]	< 0.001
Muscular strength	66 (13)	68 (13)	2 (11)	61 (18)	68 (14)	6 (15)	−1.52 [−2.16 to −0.87]	< 0.001
Total score	70 (7)	71 (7)	1 (6)	68 (8)	71 (7)	3 (6)	−1.44 [−1.75 to −1.13]	< 0.001

CI, confidence interval, SD, standard deviation. BMI, body mass index. Within-group changes for baseline, after and mean changes are expressed as mean (SD). Mean difference for the comparison between the study group and historical control was calculated by the linear mixed models and expressed as mean difference [95% CI]. The score is based on grading standards for Chinese university students. The total score is the mean of all other fitness scores for males and females. *P* < 0.05 is considered statistically significant.

### Aerobic and anaerobic fitness

In the follow-up period compared to the baseline, the study group experienced a 0.5% decrease in the 50-m run performance. Notably, this decrease was more pronounced in men, with a 0.8% decline, compared to women who showed a 0.4% decrease (Table 2). In contrast, over the same period, the mean time for the 50-m run decreased by 0.09 s for males and 0.05 s for females. The difference in the changes between the two groups was statistically significant 0.07 (95% CI: 0.04 to 0.10) (*P* < 0.001). These results indicate a decline in anaerobic fitness in the study group, while the historical control group exhibited an increase in anaerobic fitness (Fig. 3).

The decline in aerobic fitness, as indicated by changes in middle-distance run performance, was more prominent in the study group. In the study group, the mean time for the 1000-m run increased by 2.41 s, whereas in the control group, it decreased by 3.88 s, resulting in a significant between-group difference of 4.43 s (95% CI: 1.4 to 7.46) (*P* = 0.004). This suggests a decrease in aerobic fitness of approximately 2% in males and 4.7% in females in the study group (Fig. 3). Similarly, for the 800-m run, the mean time decreased by 1.95 s in the control group, while it increased by 4.22 s in the study group, resulting in a significant between-group difference of 7.36 s (95% CI: 6.33 to 8.38) (*P* < 0.001). Furthermore, the study group exhibited a greater decline in aerobic fitness as a proportion of the population compared with the control group, which showed a greater increase in aerobic fitness (Fig. S1).

### Muscular strength

Contrary to the effects on aerobic and anaerobic fitness, the impact of the lockdown on upper body strength was not consistent. Among male participants, the study group demonstrated a slight improvement in the number of achieved 1-min pull-ups, with a positive difference between baseline and follow-up indicating a performance increase of 3.33%. In contrast, the control group exhibited a negative change of 24.29%. The between-group difference was significant, with a value of 2.16 (95% CI: 1.58 to 2.74) (*P* < 0.001). For female participants, the study group showed an increase in the number of completed sit-ups in 1 min, resulting in a change of 5.65%. However, this increase was smaller than that observed in the control group (11.67%). When examining fitness change as population proportions (Fig. S1), it was evident that the study group experienced a lower decrease in fitness levels compared with the control group, although there was a greater increase in this fitness measure as a proportion in the control group.

### Explosive fitness

Regarding explosive fitness, the standing long jump test revealed a significant difference between the study and control groups. Participants in the study group had a mean jump distance of 176.51 cm (SD: 25.05), which increased by 0.71 cm (0.4%) after a year. In contrast, the control group had a mean jump distance of 175.98 cm (SD: 25.8), which increased by 4.82 cm (2.7%). The difference between the two groups was −3.43 (95% CI: −4.07 to −2.79), indicating that the control group experienced a greater increase in jump distance (*P* < 0.001). This trend was consistent for females and males (Table 2) and was reflected by a greater increase in explosive fitness in the control group (Fig. 3).

### Flexibility and vital capacity

In the sit-and-reach test, a greater improvement was observed in the study group, indicating enhanced flexibility. The mean change in the study group was 2.8 (SD: 5.05), corresponding to a 17% increase, while the control group showed a mean change of 1.36 (SD: 4.75) and an increase of 8.5%. The between-group difference was statistically significant, with a mean difference of 1.5 cm (95% CI: 1.26 to 1.73, *P* < 0.001). These results were consistent among females and males. Regarding vital capacity, no significant difference was observed between the groups, although there was a slight tendency towards a better performance in the study group.

### Weight and BMI

Regarding changes in BMI, a statistically significant difference was observed between the study group and the control group, with a between-group difference of 0.24 kg/m^2^ (95% CI: 0.17 to 0.32, *P* < 0.001). The observation group exhibited a small overall decrease in BMI (0.1%), while the historical control showed a more significant decrease (1.8%). Among females, the study group and the historical control group experienced a decrease in BMI (0.5% and 1.6%, respectively). In contrast, male participants in both groups showed an increase in weight, with the study group exhibiting a greater increase compared with the control group (1.8% increase and 0.1% increase, respectively).

### Sedentary time and exercise habits

In Fig. S2A, the mean sedentary time (in h/day) for all participants in the study group are presented for three periods. A significant increase was observed in both of these measures from the first assessment (prior to lockdown) to the second assessment (during the lockdown), as well as an increase from the second assessment to the third assessment (follow-up). In Fig. S2B and C, the changes in habitual exercise at two time points (before lockdown and follow-up) are illustrated. A decrease in exercise frequency, as well as in aerobic and strength training, was observed between the two time points.

## Discussion

Our study presents a novel approach to assess the impact of the COVID-19 pandemic on physical fitness. A historical control study design was used to investigate the longitudinal changes in fitness in a large population of young adults, a year after the onset of the pandemic. By comparing these changes to robust historical controls, strong evidence was provided regarding the effects of COVID-19 on the fitness levels of young adults. Our findings indicate that the pandemic-induced lockdown significantly undermined several dimensions of physical fitness, including aerobic and anaerobic capacities, explosive power, and weight, a year after the lockdowns. These changes have important implications for health, as they are associated with an increased risk of chronic conditions such as cardiovascular disease and type 2 diabetes, as well as heightened premature mortality risk^24,25^.

Preliminary studies on COVID-19 mitigation strategies have highlighted immediate effects on weight and psychological health. Subsequent studies have suggested that increased sedentary behaviour and reduced physical activity could result in a population-wide fitness decline^26,27^. For instance, research has demonstrated that confinement measures and the suspension of physical education classes could result in decreased cardiorespiratory fitness among adolescent elite football players, with oxygen consumption decreasing by up to 9%. Similarly, in children, these measures have been associated with significant weight gain and reduced cardiorespiratory fitness^10,28^. The closure of fitness facilities and limitations on outdoor activities are significant barriers to maintaining physical activity levels, particularly for adults who might have fewer resources and opportunities to engage in physical activity. These findings are concerning not only for younger populations experiencing diminished fitness but also for adults and older adults who might lack structured exercise routines or face greater challenges in staying physically active.

Building upon existing evidence, our study confirms notable discrepancies in weight and fitness trajectories between lockdown-affected and unaffected groups. Among males subjected to lockdown, a pronounced average weight gain of 1.3 kg was observed, despite increased activity post-lockdown, compared with a 0.7 kg gain in the control group. Although female participants from both groups experienced a decrease in annual weight, the decline was more significant among those unaffected by lockdowns. Moreover, significant declines in aerobic and anaerobic fitness were documented, along with lower body explosive fitness, 8 months after the relaxation of pandemic restrictions. These findings contrast with the improvements observed in these fitness measures among the historical control group during the same period. While adults might recover from temporary shifts in BMI and fitness as a result of resuming regular physical movement and dietary habits, this might not apply to a substantial subset of the population, particularly those lacking regular or mandatory exercise regimens.

The decline in fitness observed in our study is potentially due to a combination of disrupted physical activity routines, altered dietary habits, and pandemic-induced psychological stressors. Measures implemented to mitigate the spread of COVID-19 resulted in a significant disruption of daily life, including the closure of recreational and exercise facilities, which in turn led to reduced physical activity levels and increased sedentary behaviour^13,29^. This effect was particularly pronounced in areas with higher deprivation levels and among individuals who were previously inactive^6,30^. Concurrently, the pandemic-related stress and anxiety further exacerbated sedentary behaviours among young adults^31^. Interestingly, our study revealed a slight increase in lower body explosive fitness, as assessed by the standing long jump, within the study group affected by the pandemic. However, this increase was significantly lower than the standard reached by the control, aligning with trends observed in other studies, albeit with smaller and younger populations. Prolonged periods of sedentary behaviour, such as sitting, significantly reduce energy expenditure and muscle activation^32^, potentially leading to disuse atrophy^33^ characterised by a significant loss of skeletal muscle mass due to inactivity. These cumulative effects of increased inactivity and sedentary behaviour likely contribute to the poorer performance of the study group in fitness tests requiring lower body muscle activation^34^, such as the 100-m sprint, middle-distance run, and long jump.

Post-lockdown, upper-body strength was resilient, with male participants, in particular, showing improvements that surpassed the historical control group. Female participants also experienced some improvement, albeit lesser than the control group. This indicates a lockdown-induced shift towards resistance and body-weight exercises, particularly among males, possibly influenced by limited mobility and exercise preferences favouring intense strength training^35^. Research conducted in the United Kingdom indicated persistently low physical activity levels post-lockdown^36^. Our study expands on this finding by demonstrating that while sedentary behaviours returned to pre-pandemic levels, exercise habits remained low, which likely contributed to enduringly low cardiorespiratory and anaerobic fitness levels 8 months post-lockdown.

The findings of our study, focusing on young adults, extend to older populations with even greater significance. Older adults typically exhibit lower baseline fitness levels, and their ability to regain physical capacity after sedentary periods is often slower and more challenging due to age-related physiological changes and comorbidities^37^. Moreover, the detrimental effects of sedentary behaviour, such as insulin resistance and muscle atrophy, might manifest more acutely and rapidly in older adults, increasing their susceptibility to chronic conditions and functional decline^38^. Therefore, the potential for increased weight gain and decreased physical fitness during prolonged periods of inactivity, as indicated by our findings, could further exacerbate health risks and impede functional recovery in this demographic.

## Limitations

This study has limitations. First, despite our analysis suggesting a connection between reduced physical activity during lockdown and fitness and weight changes, further research is necessary to compare these findings with individuals who were unaffected by the lockdown. Second, it is important to note that our study focused on a large group of Han Chinese young adults, which might limit the generalisability of our findings to other populations. Lastly, while our historical control group from the same two universities shared similar age and weight baseline characteristics, some inherent differences between the two groups might have persisted. Furthermore, while we accounted for schools, age, sex, location, regional disposable income, and the value of the outcome and at baseline difference, it is important to note that the retrospective observational study design has inherent limitations, which means that complete elimination of resulting bias may not be feasible. However, considering the available options, this historical control group provided the most comparable basis for our analysis.

## Conclusion

This study provides relatively strong evidence that the COVID-19 pandemic and its mitigation measures significantly affected various aspects of physical fitness in young adults. These effects persist even a year after the implementation of lockdowns. The findings underscore the importance of continued efforts to promote physical activity during and beyond pandemics to prevent long-term detrimental consequences on health.

### Supplementary Information

Supplementary Information 1.

Supplementary Information 2.

## Data Availability

All data generated or analyzed during this study are included in this published article [and its supplementary information files].
