# Supplementary material for: The long-term impact of the COVID-19 pandemic on physical fitness in young adults: a historical control study
Source: Sci Rep. 2023 Sep 18;13:15430. doi: 10.1038/s41598-023-42710-0 (PMC10507106; doi:10.1038/s41598-023-42710-0)
Supplement: Supplementary file 1 — Supplementary Information 1. [file 41598_2023_42710_MOESM1_ESM.pdf]

Supplementary Appendix for:

The Long-term Impact of the COVID-19 Pandemic on Physical Fitness in Young Adults: A Historical Control Study

Jeffrey W. Ripley-Gonzalez[1], Nanjiang Zhou[1], Tanghao Zeng[1], Baiyang You[1,2], Wenliang Zhang[1,2], Jie Liu[3], Yuchen Dong[4], Ying Guo[3], Yaoshan Dun[1,2,5]*, Suixin Liu[1,2]*

1 Division of Cardiac Rehabilitation, Department of Physical Medicine & Rehabilitation, Xiangya Hospital of Central South University, Changsha, Hunan, China
2 National Clinical Research Center for Geriatric Disorders, Xiangya Hospital of Central South University, Changsha, Hunan, China
3 Hunan Traditional Chinese Medical College, Zhuzhou, Hunan, China
4 Medical College of Jinhua Polytechnic, Jinhua, Zhejiang, China
5 Division of Preventive Cardiology, Department of Cardiovascular Medicine, Mayo Clinic, Rochester, MN, USA

*** Correspondence:** liusuixin@csu.edu.cn (S.L.); dunyaoshan@csu.edu.cn (Ys.D.)

Running title: Impact of the COVID-19 Pandemic on Adult Fitness

Table of Contents

Supplementary Figures

Supplementary Figure 1 3

Supplementary Figure 2 4

Supplementary Table **5-8**

STROBE checklist **9-13**

Supplementary Figures

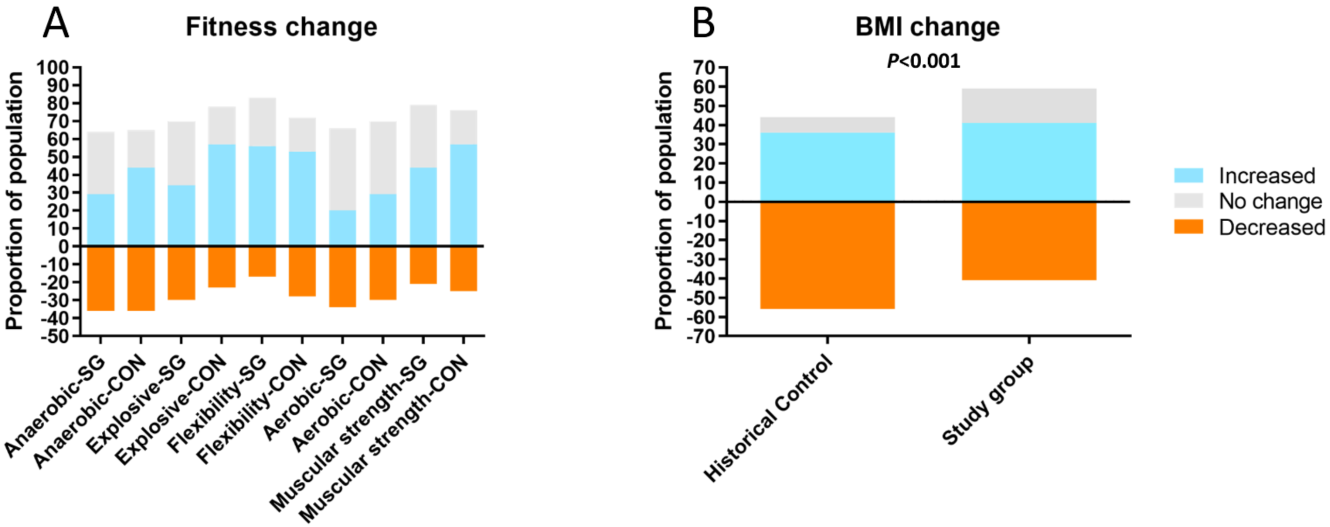

Figure S1. Fitness changes and changes in body mass index as a proportion of the population.

Figure S1A, Changes in fitness, Figure S1B changes in BMI SG- denotes the study group; CON denotes the historical control. BMI. Body mass index.

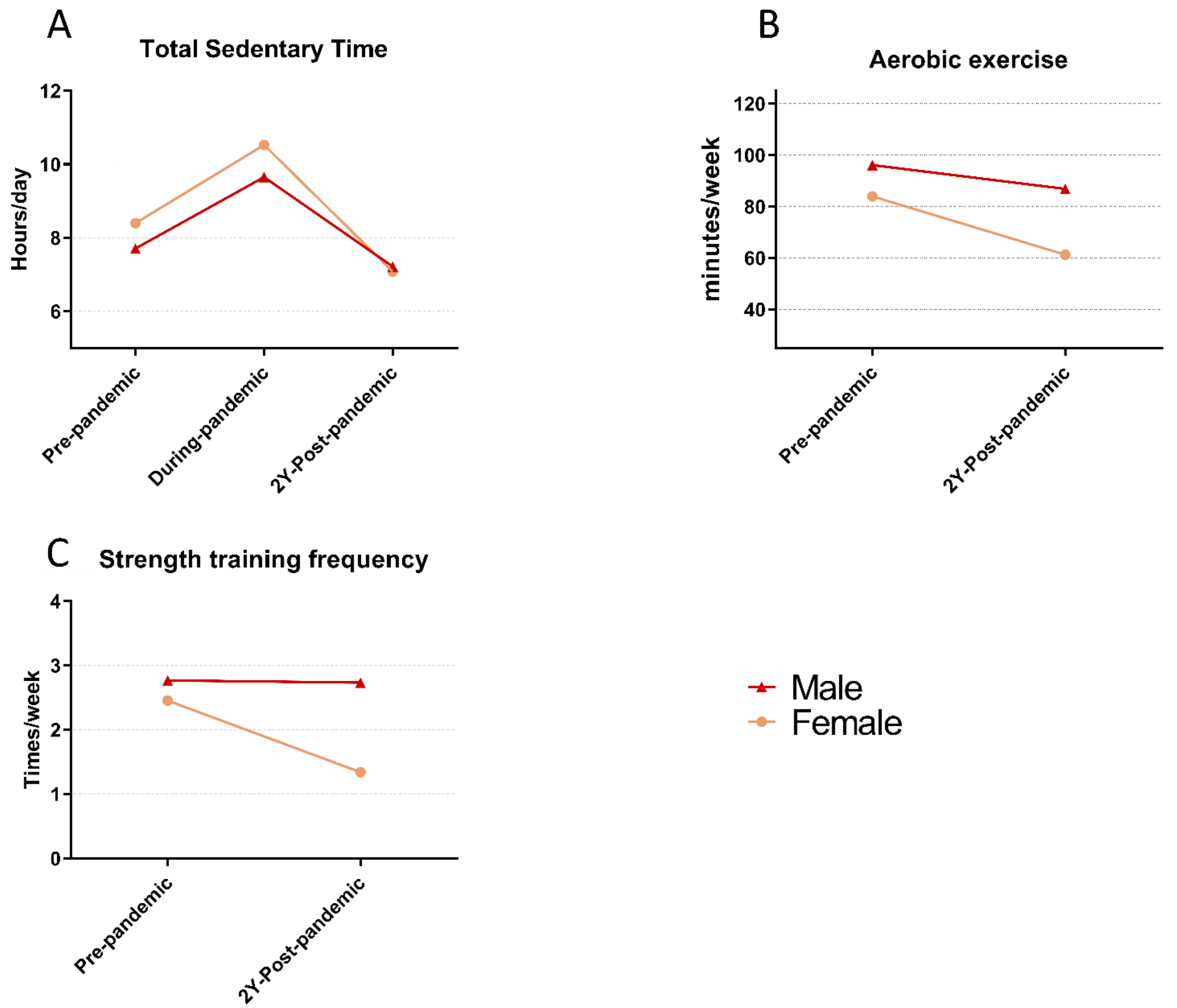

Figure S2. Sedentary and exercise habits of the Study Group.

Pre-pandemic sedentary and exercise habits were recorded at the time of the first Chinese National Student Physical Fitness Standard (CNSPFS), the during-pandemic sedentary time was recorded during lockdowns. The final recording took place one year following the initial recording. A. Shows the changes in mean sedentary time before, during and two years since the start of the COVID-19 pandemic B. Shows the changes in recorded minutes of aerobic exercise a week before and 2 years following the pandemic. C. Shows the changes in recorded minutes in the frequency (days) of strength training per week before and 2 years following the beginning pandemic.

Table S1. Subgroup analyses conducted for urban and rural subgroups to examine the consistency of the primary outcome across different areas.

**URBAN**	Study Group N=1378			Historical Control N=2024				
	Baseline mean (SD)	AFTER	mean change (SD)	Baseline mean (SD)	AFTER	mean change(SD)	Mean difference [95%CI]	*P* Value
Height (cm)	162 (7)	162 (7)	0.06 (1.46)	161.96 (7)	163 (7)	0.69 (1.51)	-0.62 [-0.72 to -0.52]	<0.001
Weight (kg)	54.2 (9.2)	54.2 (9.8)	-0.03 (3.9)	54.5 (9.8)	54.3 (9.9)	-0.2 (3.8)	0.2 [-0.06 to 0.46]	0.1
BMI (kg/m^2)	20.7 (3)	20.6 (3.1)	-0.04 (1.5)	20.7 (3)	20.5 (3.1)	-0.26 (1.51)	0.22 [0.12 to 0.32]	<0.001
Vital capacity (ml)	2934 (646)	3012 (673)	78 (457)	3060 (658)	3089 (671)	30 (393)	7.42 [-17.92 to 32.76]	0.6
Total score	69 (8)	70 (8)	1 (6)	67 (10)	69 (9)	3 (8)	-1.07 [-1.48 to -0.65]	<0.001
50m (s)	8.93 (0.91)	8.98 (0.92)	0.05 (0.52)	8.99 (0.94)	8.94 (0.98)	-0.05 (0.62)	0.06 [0.03 to 0.1]	0.001
Anaerobic fitness	71 (10)	71 (10)	-0.7 (8)	70 (11)	71 (12)	0.6 (11)	-0.63 [-1.26 to 0]	0.05
long jump (cm)	176.4 (25.5)	177.1 (25.7)	0.7 (13.2)	175.3 (25.6)	180.2 (25.9)	5 (12.4)	-3.54 [-4.35 to -2.73]	<0.001
Explosive fitness	68 (14)	69 (14)	0.5 (12)	65 (16)	70 (14)	4.5 (13)	-2.76 [-3.49 to -2.02]	<0.001
Sit and reach (cm)	16.4 (5.5)	19.1 (5.6)	2.7 (5)	15.9 (6.1)	17.4 (5.6)	1.4 (4.8)	1.42 [1.12 to 1.71]	<0.001
Flexibility	76 (12)	82 (11)	5.5 (12)	75 (15)	78 (11)	3.6 (12)	2.58 [1.93 to 3.22]	<0.001
**Male**	N= 217			N=366				
1000m run (s)	249 (23.2)	251.1 (24.9)	2.1 (21.7)	251.7 (33.6)	247.2 (25.9)	-4.5 (31.9)	4.99 [1.22 to 8.76]	0.01
Aerobic fitness	62 (13)	61 (14)	-1 (13)	61 (17)	63 (14)	2 (16)	-2.24 [-4.31 to -0.17]	0.03
One-minute pull-ups	8.8 (5.3)	9.4 (5.2)	0.5 (3.6)	8.6 (5.8)	7 (5.1)	-1.59 (5.79)	2.24 [1.52 to 2.96]	<0.001
Muscular strength	45 (34)	48 (33)	3 (23)	42 (35)	33 (32)	-10 (37)	13.76 [9.12 to 18.4]	<0.001
**Female**	N=1161			N=1658				
800m run (s)	240 (19.4)	244 (20)	4.3 (17)	238 (25.7)	235.7 (20)	-2.5 (24.9)	7.88 [6.57 to 9.19]	<0.001

Aerobic fitness	67 (12)	64 (14)	-3 (13)	67 (16)	68 (12)	2 (16)	-4.33 [-5.21 to -3.45]	<0.001
One-minute sit-ups	33.2 (7.7)	35 (8.2)	1.8 (6.4)	31.4 (9.1)	35.4 (8.3)	3.9 (7.1)	-1.43 [-1.88 to -0.97]	<0.001
Muscular strength	65 (14)	67 (14)	2 (12)	60 (19)	67 (14)	7 (16)	-2.04 [-2.91 to -1.18]	<0.001

...Table S1 continued.

RURAL	Study Group N=861			Historical Control N=1113				
	Baseline mean (SD)	AFTER	mean change (SD)	Baseline mean (SD)	AFTER	mean change(SD)	Mean difference [95%CI]	*P* Value
Height (cm)	160 (6)	161 (7)	0.14 (1.45)	161 (7)	162 (7)	0.61 (1.38)	-0.44 [-0.57 to -0.32]	<0.001
Weight (kg)	53.11 (8.9)	53.16 (9.39)	0.04 (3.95)	53.46 (9.11)	53.15 (9.25)	-0.32 (3.56)	0.43 [0.1 to 0.76]	0.01
BMI (kg/m^2)	20.5 (2.94)	20.47 (3.06)	-0.03 (1.55)	20.56 (2.88)	20.27 (2.86)	-0.28 (1.4)	0.27 [0.14 to 0.4]	<0.001
Vital capacity (ml)	2875 (583)	2977 (642)	102 (477)	3058 (668)	3078 (664)	19.97 (389.59)	36.77 [3.23 to 70.32]	0.03
Total score	70 (7)	71 (8)	1 (6)	68 (9)	70 (8)	2 (6)	-0.8 [-1.32 to -0.29]	0.002
50m (s)	9 (0.9)	9.03 (0.89)	0.04 (0.53)	8.97 (0.9)	8.91 (0.94)	-0.06 (0.58)	0.08 [0.03 to 0.12]	0.001
Anaerobic fitness	71 (10)	70 (11)	-0.7 (9)	71 (10)	71 (10)	0.8 (9)	-1.17 [-1.93 to -0.42]	0.002
long jump (cm)	176.7 (24.4)	177.5 (24.5)	0.8 (13.1)	177.3 (26.1)	181.8 (26.5)	4.6 (11.9)	-3.21 [-4.25 to -2.17]	<0.001
Explosive fitness	70 (14)	70 (13)	0.6 (11)	68 (15)	71 (13.57)	4 (11)	-2.52 [-3.39 to -1.64]	<0.001
Sit and reach (cm)	16.5 (5.6)	19.4 (5.8)	2.9 (5.1)	16.4 (6.1)	17.6 (5.9)	1.2 (4.7)	1.63 [1.24 to 2.02]	<0.001
Flexibility	76 (12)	82 (12)	6 (10)	75 (15)	79 (12)	3 (13)	3.01 [2.14 to 3.87]	<0.001
**Male**	N=112			N=194				
1000m run (s)	245.5 (25.9)	248.5 (25.3)	3.1 (25.3)	249.8 (30.2)	247.1 (26)	-2.7 (27.3)	3.59 [-1.53 to 8.71]	0.2
Aerobic fitness	64 (13)	62 (14)	-2 (14)	61 (16)	63 (14)	2 (15)	-2.35 [-5.21 to 0.52]	0.1
One-minute pull-ups	9.2 (5.5)	9.2 (5.4)	-0.04 (4)	8.7 (5.5)	6.9 (5)	-1.8 (5.3)	2.01 [1.03 to 2.99]	<0.001
Muscular strength	49 (33)	48 (33)	-1 (26)	44.21 (34.25)	33 (32)	-11 (34)	11.95 [5.65 to 18.24]	<0.001
**Female**	N=749			N=919				
800m run (s)	239.2 (18.8)	243.4 (21.6)	4.1 (18.3)	235.7 (21.7)	234.9 (19.2)	-0.9 (20.1)	6.3 [4.67 to 7.93]	<0.001
Aerobic fitness	67 (12)	64 (15)	-3 (14)	68 (13)	69 (11)	0.8 (13)	-4.09 [-5.22 to -2.97]	<0.001

One-minute sit-ups	34.3 (7.2)	36.4 (7.9)	2.1 (5.9)	32.3 (8.2)	35.54 (8)	3.28 (6.4)	-0.47 [-1.03 to 0.08]	0.1
Muscular strength	67 (11)	69 (11)	3 (10)	62.5 (17)	68 (13)	5 (13)	-0.58 [-1.52 to 0.36]	0.2

CI, confidence interval, SD, standard deviation. BMI, body mass index.

Within-group changes for baseline, after and mean changes are expressed as mean (SD). Mean difference for the comparison between the study group and historical control was calculated by the linear mixed models and expressed as mean difference [95% CI]. $P < 0.05$ is considered statistically significant. The score is based on Grading Standards for Chinese university Students. Total score is mean of all other fitness scores, Aerobic fitness, Anaerobic fitness, Explosive fitness, Muscular strength, Vital capacity and flexibility for male and females.

STROBE Statement—checklist of items that should be included in reports of observational studies				
	**Item No.**	**Recommendation**	**Page No.**	**Relevant text from manuscript**
**Title and abstract**	1	(*a*) Indicate the study's design with a commonly used term in the title or the abstract	1	
		(*b*) Provide in the abstract an informative and balanced summary of what was done and what was found	2	
**Introduction**				
Background/rationale	2	Explain the scientific background and rationale for the investigation being reported	3	
Objectives	3	State specific objectives, including any prespecified hypotheses	3	
**Methods**				
Study design	4	Present key elements of study design early in the paper	3-4	
Setting	5	Describe the setting, locations, and relevant dates, including periods of recruitment, exposure, follow-up, and data collection	3-4	
Participants	6	(*a*) *Cohort study*—Give the eligibility criteria, and the sources and methods of selection of participants. Describe methods of follow-up<br>*Case-control study*—Give the eligibility criteria, and the sources and methods of case ascertainment and control selection. Give the rationale for the choice of cases and controls<br>*Cross-sectional study*—Give the eligibility criteria, and the sources and methods of selection of participants	3	
		(*b*) *Cohort study*—For matched studies, give matching criteria and number of exposed and unexposed	4-5	

		Case-control study—For matched studies, give matching criteria and the number of controls per case	
Variables	7	Clearly define all outcomes, exposures, predictors, potential confounders, and effect modifiers. Give diagnostic criteria, if applicable	4
Data sources/ measurement	8*	For each variable of interest, give sources of data and details of methods of assessment (measurement). Describe comparability of assessment methods if there is more than one group	3-4
Bias	9	Describe any efforts to address potential sources of bias	8
Study size	10	Explain how the study size was arrived at	4

Continued on next page

Quantitative variables	11	Explain how quantitative variables were handled in the analyses. If applicable, describe which groupings were chosen and why	4
Statistical methods	12	(*a*) Describe all statistical methods, including those used to control for confounding	4
		(*b*) Describe any methods used to examine subgroups and interactions	4
		(*c*) Explain how missing data were addressed	4
		(*d*) *Cohort study*—If applicable, explain how loss to follow-up was addressed<br>*Case-control study*—If applicable, explain how matching of cases and controls was addressed<br>*Cross-sectional study*—If applicable, describe analytical methods taking account of sampling strategy	4
		(*e*) Describe any sensitivity analyses	4
**Results**			
Participants	13*	(a) Report numbers of individuals at each stage of study—eg numbers potentially eligible, examined for eligibility, confirmed eligible, included in the study, completing follow-up, and analysed	5
		(b) Give reasons for non-participation at each stage	Figure 1
		(c) Consider use of a flow diagram	Figure 1
Descriptive data	14*	(a) Give characteristics of study participants (eg demographic, clinical, social) and information on exposures and potential confounders	Table 1
		(b) Indicate number of participants with missing data for each variable of interest	N/A
		(c) *Cohort study*—Summarise follow-up time (eg, average and total amount)	5
Outcome data	15*	*Cohort study*—Report numbers of outcome events or summary measures over time	N/A
		Case-control study—Report numbers in each exposure category, or summary measures of exposure	N/A
		Cross-sectional study—Report numbers of outcome events or summary measures	N/A
Main results	16	(*a*) Give unadjusted estimates and, if applicable, confounder-adjusted estimates and their precision (eg, 95% confidence interval). Make clear which confounders were adjusted for and why they were included	5
		(*b*) Report category boundaries when continuous variables were categorized	4

	(*c*) If relevant, consider translating estimates of relative risk into absolute risk for a meaningful time period	N/A

Continued on next page

Other analyses	17	Report other analyses done—eg analyses of subgroups and interactions, and sensitivity analyses	5-6
**Discussion**			
Key results	18	Summarise key results with reference to study objectives	6
Limitations	19	Discuss limitations of the study, taking into account sources of potential bias or imprecision. Discuss both direction and magnitude of any potential bias	7
Interpretation	20	Give a cautious overall interpretation of results considering objectives, limitations, multiplicity of analyses, results from similar studies, and other relevant evidence	7
Generalisability	21	Discuss the generalisability (external validity) of the study results	7
**Other information**			
Funding	22	Give the source of funding and the role of the funders for the present study and, if applicable, for the original study on which the present article is based	10

*Give information separately for cases and controls in case-control studies and, if applicable, for exposed and unexposed groups in cohort and cross-sectional studies.
