# Supplementary figures and images for: The long-term impact of the COVID-19 pandemic on physical fitness in young adults: a historical control study
Source: Sci Rep. 2023 Sep 18;13:15430. doi: 10.1038/s41598-023-42710-0 (PMC10507106; doi:10.1038/s41598-023-42710-0)

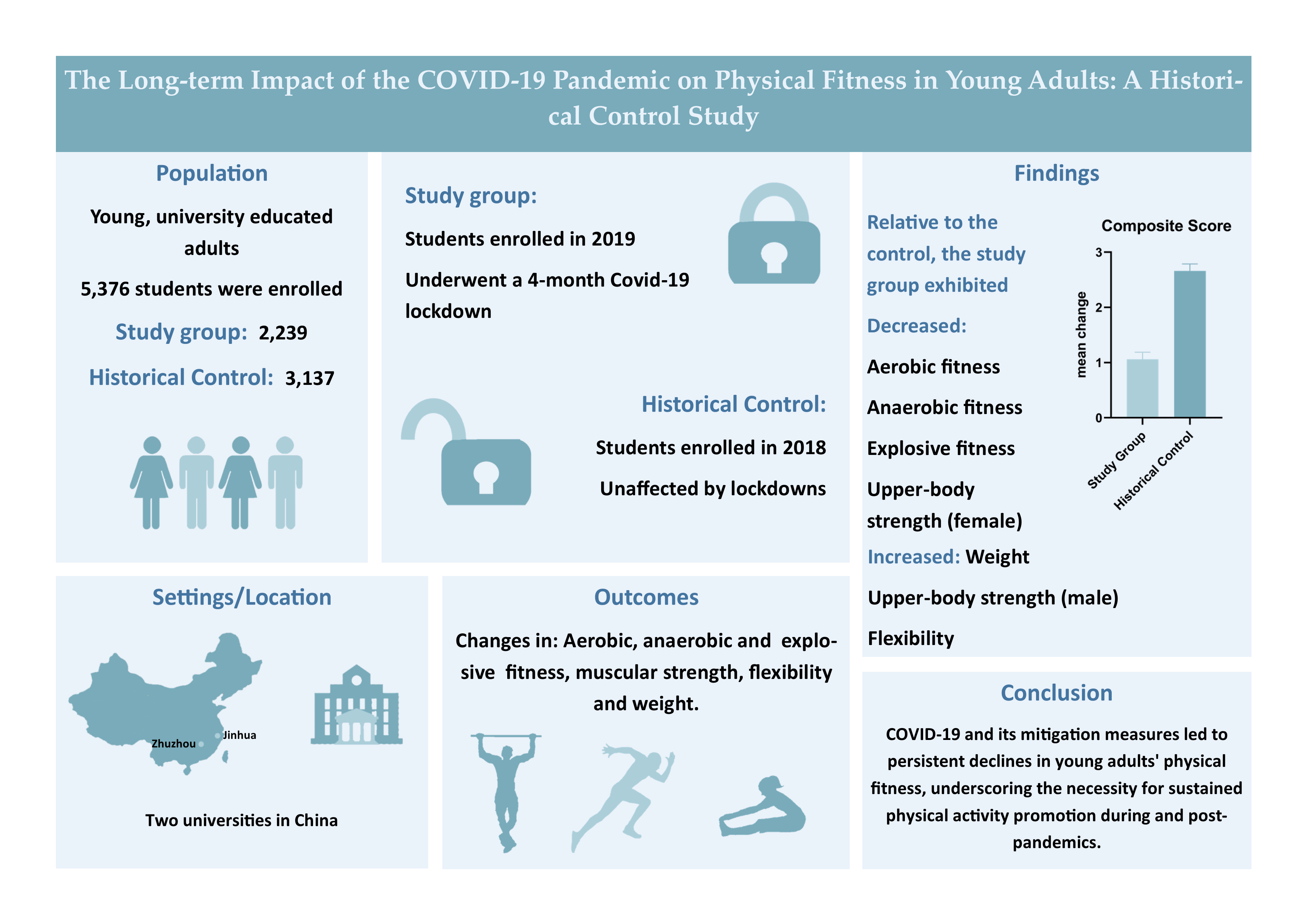

Supplement: Supplementary file 2 — Supplementary Information 2. [file 41598_2023_42710_MOESM2_ESM.tif]
